# Pathological Site Pain During Injections as a Predictive Sign for Clinical Response in Autologous Protein Solution and Hyaluronic Acid Injections for Knee Osteoarthritis

**DOI:** 10.1016/j.jcot.2024.102901

**Published:** 2024-12-31

**Authors:** Edmund Jia Xi Zhang, Craigven Hao Sheng Sim, Zachariah Gene Wing Ow, Edward Vincentius Lie, Krishmen Rasu, Keng Lin Wong

**Affiliations:** a Yong Loo Lin School of Medicine, National University of Singapore, Singapore, Singapore; b Department of Orthopaedic Surgery, Sengkang General Hospital, Singapore; c Department of Orthopaedic Surgery, National University Hospital, Singapore

**Keywords:** Autologous protein solution, Knee osteoarthritis

## Abstract

**Objectives:**

Autologous peripheral blood-derived orthobiologics like platelet-rich plasma (PRP) have been gaining in popularity in symptomatic relief of knee osteoarthritis (OA). Autologous protein solution (APS) that is derived from PRP offers higher levels of growth factors and anti-inflammatory cytokines, reducing inflammation and improve cartilage quality. Additionally, hyaluronic acid (HA) has shown efficacy in relieving OA symptoms. This study aims to assess the clinical outcomes of combined APS and HA therapy, particularly a presence of pathological site pain (PSP) during injection as a predictive sign for clinical response.

**Methods:**

Patients with early-stage OA received APS and HA injections. Patients were evaluated pre-injection and at 1-year follow-up. Patient-reported outcomes were assessed with WOMAC, KOOS, VAS pain score, and SF-36 survey. The OMERACT–OARSI criteria determined treatment effects. Satisfaction and expectation fulfillment were also recorded.

**Results:**

32 patients were included in the final analysis. Statistically significant improvements were observed in all outcome scores at 1 year. The responder rate per OMERACT-OARSI criteria was 65.6 %, with 96.9 % of patients reporting satisfaction and expectation fulfillment. When comparing responder-rates and improvement in patient-reported outcome measures with other studies, combined therapy does not appear to confer additional therapeutic benefit over APS monotherapy at the 1-year mark. No severe adverse events related to the injections were reported. Patients with PSP had significantly better outcomes in terms of pain, stiffness, symptoms, activities of daily living, quality of life, as well as statistically significantly higher satisfaction rates of expectation fulfilment.

**Conclusions:**

At 1-year post-injection, the APS and HA combination significantly improved WOMAC, KOOS, SF-36 PCS, and VAS scores, with a high rate of patient satisfaction. PSP during injection could possibly be predictive of better outcomes and expectation fulfilment.

**Level of evidence:**

Level III.

## 1 Introduction

Knee osteoarthritis (OA) has a significant negative impact on quality of life.^1^ Identification of therapies that improve health-related quality of life (HRQoL) in patients with knee OA may mitigate the clinical, economic, and social burden of this disease.^2^

Currently, various symptomatic therapy options, such as analgesics, nonsteroid anti-inflammatory drugs, and preparations for topical administration, are being used, with a diverse clinical response and inconsistent conclusions across guidelines.^3^ Recently, the popularity of autologous peripheral derived orthobiologics (APBOs) has been increasing,^4^^,^^5^ such as platelet rich plasma (PRP). The rationale of PRP is that concentrated platelets at the site of injury initiates tissue repair through the release of biologically active factors and adhesion proteins,^6^ and would help reduce pain and modulate the disease process.^7^

Autologous protein solution (APS) is derived from further processing of PRP, and has higher levels of growth factors and anti-inflammatory cytokines and lower levels of pro-inflammatory cytokines.^8^ Compared to the other ABPOs, APS is hypothesized to augment the anabolic effects of PRP with autologous anti-inflammatory homeostatic properties.^8^ APS contains anti-inflammatory cytokines which are antagonistic to the inflammatory cytokines IL-1β and TNFα that are largely responsible for driving cartilage degeneration in OA.^8^ In vitro studies have demonstrated that APS can reduce inflammatory markers in synovial fluid and improve cartilage quality, providing a strong scientific rationale for its clinical use.^9^^,^^10^ Preclinical studies in canine and equine models have also shown symptomatic improvement.^11^^,^^12^

Conversely, hyaluronic acid (HA) has been established as efficacious in symptomatic relief in OA knees due to its physical-chemical properties and interaction with cartilages' cells and extracellular matrixes.^13^ Studies have shown that PRP, when given together with HA, have better functional outcomes, as compared to when these components are given individually.^14^, ^15^, ^16^, ^17^, ^18^

While PRP combined with HA has demonstrated better outcomes compared to monotherapy, APS and HA presents a novel approach with potential advantages. APS offers a more concentrated anti-inflammatory profile, potentially enhancing the chondroprotective and regenerative properties of HA. Unlike PRP, APS directly antagonizes pro-inflammatory cytokines such as IL-1β and TNFα, which are implicated in cartilage degradation. This combination may provide synergistic benefits by coupling APS's anti-inflammatory properties with HA's viscosupplementation and lubrication effects. In addition, current reviews highlight the need for more studies with comprehensive reporting of outcome measures to further for APS to be widely accepted as a treatment modality for OA.^8^^,^^13^

Furthermore, in our experience, we also noted a dichotomy of immediate response in patients who experienced pain during injections. This pain was described as a reproduction of their symptomatic knee pain at the same site, and we postulated that pathological site pain (PSP) during injections possibly predicts response and patients with PSP might experience better outcomes.

Therefore, the aims of this study are to assess the clinical outcomes after a combined APS and HA therapy and assess whether PSP during injection was associated with better outcomes.

## 2 Methods

An Institutional Review Board approval (2020/2735) was granted for this retrospective study.

This is a retrospective study of prospectively collected cohort data from September 1, 2020 to April 12, 2024. Data was retrieved from the hospital's electronic medical record system. Patients diagnosed with early-stage osteoarthritis were offered APS and HA injection. Patients with pain caused by focal chondral defects and Kellgren-Lawrence stage four osteoarthritis were excluded from this study. Demographics recorded were the patient's age, gender, side of knee involvement, and body-mass index (BMI).

### 2.1 Patient evaluation

Patients were evaluated in the clinic before the injection and at the six-week, 12-week, six-month, one-year, and two-year mark. Objective measurements of the patient collected were the affected knee's flexion and extension range of motion (ROM), alignment, average mean muscle test, sit-to-stand time, and timed up-and-go test. Clinical efficacy was assessed with the following tools: Western Ontario and McMaster Universities Osteoarthritis Index (WOMAC) questionnaire and Knee injury and Osteoarthritis Outcome Score (KOOS) questions, assessments of pain with the visual analog scale (VAS) pain score, and health-related quality of life evaluated using the Short Form–36 (SF-36) survey. All objective assessments and questionnaires were administered by a trained physiotherapist.

**Fig. 1:**
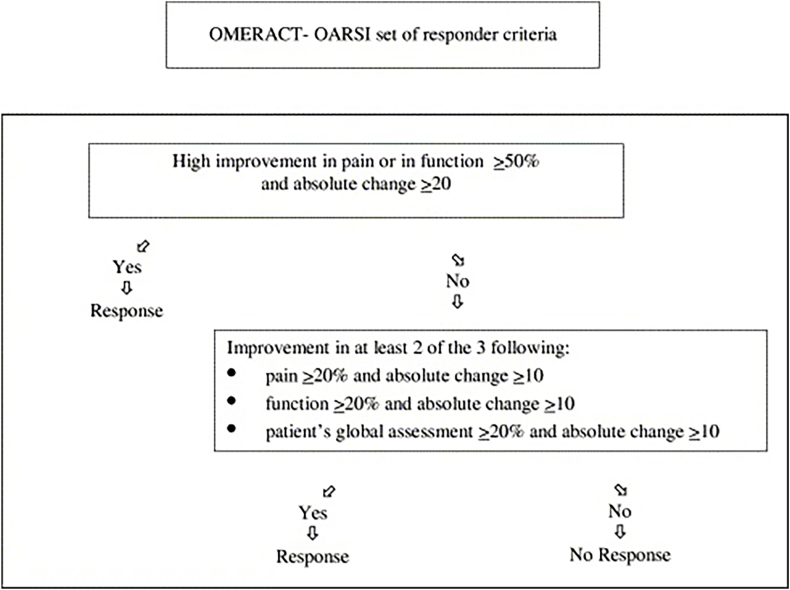
OMERACT-OARSI responder criteria.^19^.

In addition, the Outcome Measures in Arthritis Clinical Trials–Osteoarthritis Research Society International (OMERACT–OARSI) criteria^19^ were used to determine the effect of the intervention (Fig. 1).

Patients were also asked to rate the overall results of their treatment in terms of satisfaction and whether their expectations had been met between excellent, very good, good, fair, poor and terrible. Satisfaction is considered achieved and expectations met if the patient rated good, very good or excellent.

For patients who did not turn up for the one-year follow-up appointment, they were contacted via telephone. For these patients, the WOMAC, VAS, SF-36, and satisfaction and expectation questions were administered. Objective measurements were not collected.

### 2.2 Injection technique

The nSTRIDE APS kit (Biomet Biologics, Warsaw, IN, USA) was used. 60 mL of peripheral blood was drawn and mixed with Anticoagulant Citrate Dextrose Solution Formula. It was then passed through the nSTRIDE Cell Separator which separates the cellular and platelet components of whole blood to form a cell solution. It is then passed through the nSTRIDE Concentrator to be concentrated via filtration through polyacrylamide beads. Approximately 3 mL of injectable APS is collected.

The APS was then injected directly into the symptomatic knee joint, followed by hyaluronic acid Monovisc (Anika Therapeutics, Bedford, MA, USA).

### 2.3 Statistical analysis

IBM SPSS software was used to conduct the statistical analysis (IBM, version 29.0). The Wilcoxon rank-sum test was used to assess for differences among data that was not normally distributed. Differences among and between means and proportions were declared statistically significant if *P* < 0.05.

## 3 Results

From September 2020 to April 2024, a total of 101 patients diagnosed with osteoarthritis received the APS and HA injection. As of April 2024, there were 67 patients who had more than one year since the day of injection. Out of the 67 patients, 18 patients turned up for their one-year follow-up appointment, and two had knee replacement surgeries. All remaining patients were contacted via telephone, and 14 patients were followed-up via telephone.

A final total 32 patients were included in the analysis, of which 17 were female patients. The average age of these patients was 53.2 ± 8.01 years. The patient characteristics are summarized below (Table 1).

**Table 1:** Patient Characteristics

Characteristic	Values
Age, years (SD)	53.2 ± 8.01
BMI (SD)	26.5 ± 5.38
Gender	
Male	15
Female	17
Injection side	
Left	17
Right	15

There was statistically significant improvement in all outcome scores for patients at the 1 year mark (Table 2). There was an improvement of 45.9 % for VAS, 53.1 % for WOMAC, and 24.9 % for SF36 Physical Component Score.

**Table 2:** VAS, WOMAC, KOOS, and SF36 PCS Scores

	Pre-APS	Post-APS (12 months)	p value
**VAS**	3.64 ± 2.01	1.97 ± 1.39	<0.001
**WOMAC**			
Pain	71.8 ± 20.0	85.5 ± 18.0	<0.001
Stiffness	72.8 ± 32.2	88.6 ± 17.1	0.003
Physical Activity	76.0 ± 20.2	89.0 ± 15.5	<0.001
**KOOS**			
Pain	67.4 ± 18.5	82.4 ± 17.3	<0.001
Symptoms	69.5 ± 21.1	85.8 ± 16.4	<0.001
Activities of daily living	77.7 ± 18.4	87.7 ± 15.5	0.001
Sport and Recreation/Activities	37.8 ± 25.7	61.5 ± 31.6	<0.001
Quality of life	47.0 ± 18.7	71.1 ± 25.1	<0.001
**SF36 PCS**	38.2 ± 9.21	47.7 ± 9.87	<0.001

VAS: Visual Analogue Scale; WOMAC: Western Ontario and McMaster Universities Osteoarthritis Index; KOOS: Knee Injury and Osteoarthritis Outcome Score; SF36 PCS: 36-Item Short Form Survey Physical Component Score.

96.9 % of patients reported being satisfied with the injection and having their expectations met (Table 3). The overall responder rate under the OMERACT-OARSI criteria was 65.6 % (Table 4).

**Table 3:** Fulfilment of Expectations and Satisfaction at 12 Months

**Expectations**	

Fulfilled	96.9 %
Not fulfilled	3.1 %

**Satisfaction**	

Fulfilled	96.9 %
Not fulfilled	3.1 %

**Table 4:** Responder Rate at 12 Months

Response	65.6 %
No Response	34.4 %

11 patients reported PSP during the injection. None of the patients reported any injection site infections, bleeding, or numbness that were considered related to the injection. Patients with PSP had significantly better outcomes in terms of pain, stiffness, symptoms, activities of daily living, quality of life, as well as statistically significantly higher satisfaction rates of expectation fulfilment (Table 5). While patients with PSP fared better for all other outcomes, these improvements were not clinically significant.

**Table 5:** 12 Month Outcomes for PSP and Non-PSP

	PSP	Non-PSP	*p* value
**VAS**	1.73 ± 1.57	2.03 ± 1.24	0.404
**WOMAC**			
Pain	90.2 ± 15.6	83.1 ± 19.0	0.147
Stiffness	98.6 ± 3.23	83.3 ± 19.1	0.007
Physical Activity	93.9 ± 14.0	86.4 ± 16.0	0.101
**KOOS**			
Pain	87.6 ± 14.0	78.9 ± 18.5	0.093
Symptoms	93.7 ± 7.92	81.0 ± 18.4	0.019
Activities of daily living	95.3 ± 9.77	82.9 ± 16.6	0.016
Sport and Recreation/Activities	71.1 ± 30.6	55.4 ± 32.0	0.097
Quality of life	84.1 ± 22.6	62.8 ± 23.7	0.021
**SF36 PCS**	50.6 ± 10.1	46.1 ± 9.61	0.117
**Expectations**			
Fulfilled	100.0 %	71.4 %	0.049
**Satisfaction**			
Fulfilled	100.0 %	95.2 %	0.462
**Responder Status**	81.8 %	57.1 %	0.163

## 4 Discussion

The main findings of this study demonstrate that a combined intra-articular injection of APS and HA provides significant pain and symptom relief lasting at least 12 months, and that PSP may be associated with better outcomes and expectation fulfilment.

Viscosupplementation in the form of hyaluronic acid helps to restore the physiologic viscoelasticity in the synovial fluid.^20^ Hyaluronic acid is a vital component of synovial fluid and is essential for proper lubrication of joints^21^ and has been shown to exhibit chondroprotective effects.^22^, ^23^, ^24^ Studies, including a Cochrane review by Bellamy et al.,^25^ have shown that intra-articular hyaluronic acid injections have provided symptomatic relief and improvement in physical function.

Our results are congruent with the few studies that have assessed the effects of APS beyond 1 year. At 1 year follow-up, Kuwasa et al.^26^ and Kon et al.^20^ showed that APS provided significant pain improvement for knee OA. Kon et al.^21^ also showed that significant improvement was maintained at three years, and postulated that APS may contribute to more than temporary symptomatic relief, potentially slowing the progression of OA-related structural changes and chronic disability.

Studies have shown favourable effects of combining PRP and HA that are superior to when these components are given individually.^15^^,^^17^^,^^29^^,^^30^ It has been suggested that the viscosupplementation and elastic properties, when combined with the potential chondroprotective effects of PRP, may facilitate the activity of inflammatory molecules, cytokines, and catabolic enzymes.^24^ Currently, this has been investigated in canine and mice models, which has shown increased glycosamingoclycan content, decreased apoptosis, and reduced cartilage damage.^27^, ^28^, ^29^

PRP contains concentrated platelets, while APS contains plasma proteins with elevated cytokine levels. APS has been shown to potentially maintain joint function by blocking matrix metalloprotease production,^9^ inflammatory cytokines production,^30^ and extracellular matrix degradation in inflammatory conditions.^10^

The findings of our study align with the growing body of literature exploring the clinical applications of APS in knee osteoarthritis.^5^^,^^13^ However, the results of a recent study comparing APS to saline suggest limited efficacy of APS monotherapy.^31^ Specifically, no significant differences were observed in WOMAC or KOOS scores over 12 months, and APS-treated patients reported worse VAS pain scores compared to saline. This surprising result highlights the limitations of APS monotherapy and the need to explore enhanced therapeutic strategies.

The combined use of APS and HA may address these limitations by capitalizing on their complementary effects. While APS provides anti-inflammatory and regenerative benefits, HA contributes to mechanical lubrication and viscosupplementation. These combined properties may offer a more holistic approach to managing knee OA, addressing both inflammation and biomechanical dysfunction. Although our study did not directly compare APS + HA to APS monotherapy, the significant improvements observed in WOMAC, KOOS, and VAS scores at 12 months suggest that combining APS with HA may help overcome the challenges identified in previous trials of APS alone. In our study, this combination was able to elicit significant improvements in all patient-reported outcomes with high satisfaction and expectation fulfilment at the 1-year mark.

Currently, there are no studies comparing the effectiveness of APS monotherapy and combined APS and HA injection. When comparing response rates based on the OMERACT-OARSI criteria at the one-year mark, our study of combined injection had a responder rate of 65.6 %, as compared to 55 % in Kuwasawa et al.^26^ and 65.5 % in Kon et al.,^20^ both of which gave solely APS. WOMAC score improvement was 53.1 % in our study and 65 % in Kon et al.,^20^ and VAS score improvement was 45.9 % in our study and 49 % in Kon et al.^20^

In our study, improvement in WOMAC scores have achieved minimal clinically important difference based on values described by Holtz et al.^32^ and Clement et al.^33^ However, when comparing responder-rates and improvement in patient-reported outcome measures, combined therapy of APS and HA does not appear to confer additional therapeutic benefit over APS monotherapy at the 1-year mark.

The lack of additional benefit despite addition of HA might be due to the limited duration of effect of viscosupplementation. Different studies have described the mean duration of effect to be less than 12 months.^34^^,^^35^ The temporary effect of intra-articular viscosupplementation could be due to the turnover of hyaluronic acid and removal of synovial fluid, which has been demonstrated in rabbit models.^36^ Lindqvist et al.^37^ demonstrated the elimination kinematics of hyaluronic acid in healthy males, with elimination half-lives to 4 weeks. Hence, while HA might confer additional potential benefits, these improvements over APS monotherapy are unlikely to last beyond the 1-year mark.

There is currently no established explanation for PSP during intra-articular injection. We postulate that it could be due to remnant surviving chondrocytes at the pathological site releasing pro-inflammatory cytokines in response to the injection. This acute inflammatory signaling may transiently heighten nociceptive activity, causing immediate pain. Importantly, this acute pain could reflect a heightened biological activity at the pathological serves, which possibly serves as a precursor to subsequent reparative mechanisms.

The high concentrations of growth factors, cytokines, and other bioactive molecules in APS likely amplify this acute inflammatory response. These elements not only counteract inflammatory mediators but also set the stage for repair processes, such as enhanced extracellular matrix production and decreased catabolic activity.

These findings suggest that PSP might act as a possible predictor for the efficacy of APS and HA treatment. This is important in clinical practice, where injection-related pain is often viewed negatively by patients and clinicians. Recognizing that immediate pain does not indicate a poor prognosis but instead may predict better outcomes and expectation fulfilment allows clinicians to better manage and counsel patients regarding PSP. Future higher-powered research should explore this phenomenon and elucidate the possible mechanism behind it. Additionally, imaging studies could explore whether PSP is associated with localized synovial changes or cartilage regeneration, providing a more detailed understanding of its biological significance.

The absence of severe adverse events related to the injections in our study suggests that combined APS and HA injection is safe. This is in line with other studies that have reported the safety profile of APS.^38^ Studies done on rats, canines, and horses have also concluded on the safety of APS and did not find any evident association with adverse events.^11^^,^^12^^,^^39^ Potential mild adverse effects reported in other studies include arthralgia, joint effusion, stiffness, injection site pain and discomfort, and procedure-associated nausea.^40^

### 4.1 Limitations

Our study has limitations that warrant consideration. The small sample size may limit the statistical power and generalizability of the findings. The absence of active comparator groups, such as APS or HA monotherapy, is another limitation of this study. However, with the modest improvements with APS monotherapy^31^ and transient relief with HA, our aim was to assess the combined benefits. In addition, there was no imaging or biomarker studies to validate the proposed mechanism of PSP and its relationship to outcomes. Further larger studies with longer follow-up periods are needed, augmented with imaging or biomarker investigations.

To build on these results, future research should focus on confirming the efficacy of combined HA and APS injections through larger, randomized controlled trials with regular follow-up durations to determine its effective duration. These studies should also aim to elucidate the specific mechanisms through which APS and HA exert their therapeutic effects and assess for PSP, possibly with imaging or biomarker investigations.

## 5 Conclusion

In conclusion, at 12 months post-injection, a combined protocol of APS and HA significantly improved WOMAC, KOOS, SF36 PCS and VAS scores, with high patient satisfaction rate. However, the combined protocol does not seem to confer additional benefit over APS monotherapy at 12 months.

## CRediT authorship contribution statement

**Edmund Jia Xi Zhang:** Data curation, Writing – original draft, prepartion. **Craigven Hao Sheng Sim:** Writing – Reviewing and Editing. **Zachariah Gene Wing Ow:** Writing – Reviewing and Editing. **Edward Vincentius Lie:** Writing – Reviewing and Editing. **Krishmen Rasu:** Methodology, Data curation. **Keng Lin Wong:** Conceptualization, Writing – Reviewing and Editing, Supervision.

## Patient informed consent statement

The author(s) should confirm that written informed consent has been obtained from the involved patient(s) or if appropriate from the parent, guardian, power of attorney of the involved patient(s); and, they have given approval for this information to be published in this case report (series).

Complete written informed consent was obtained from the patient for the publication of this study and accompanying images.

## Guardian/patient's consent

Patient consent was obtained.

All analyses were performed in compliance with relevant laws and institutional guidelines and have been approved by the appropriate institutional committee(s).

## Ethical approval

An Institutional Review Board approval (2020/2735) was granted for this retrospective study.

## Funding statement

This research did not receive any specific grant from funding agencies in the public, commercial, or not-for-profit sectors.

## Declaration of competing interest

The authors declare that they have no known competing financial interests or personal relationships that could have appeared to influence the work reported in this paper.
